# Azilsartan Modulates HMGB1/NF-κB/p38/ERK1/2/JNK and Apoptosis Pathways during Renal Ischemia Reperfusion Injury

**DOI:** 10.3390/cells12010185

**Published:** 2023-01-02

**Authors:** Rania Alaaeldin, Sally M. Bakkar, Reham H. Mohyeldin, Fares E. M. Ali, Nehad M. Reda Abdel-Maqsoud, Moustafa Fathy

**Affiliations:** 1 Department of Biochemistry, Faculty of Pharmacy, Deraya University, Minia 61519, Egypt; 2 Department of Biochemistry, Faculty of Medicine, Assiut University, Assiut 71515, Egypt; 3 Department of Pharmacology and Toxicology, Faculty of Pharmacy, Deraya University, Minia 61519, Egypt; 4 Department of Pharmacology and Toxicology, Faculty of Pharmacy, Al-Azhar University, Assiut Branch, Assiut 71524, Egypt; 5 Department of Pathology, Faculty of Medicine, Deraya University, Minia 61519, Egypt; 6 Department of Biochemistry, Faculty of Pharmacy, Minia University, Minia 61511, Egypt; 7 Department of Regenerative Medicine, Graduate School of Medicine and Pharmaceutical Sciences, University of Toyama, Toyama 930-0194, Japan

**Keywords:** azilsartan, renal ischemia/reperfusion injury, NF-κB, ERK1/2, JNK, apoptosis

## Abstract

Renal ischemia/reperfusion (IR) injury is characterized by an unexpected impairment of blood flow to the kidney. Azilsartan is an angiotensin receptor blocker that is approved for the management of hypertension. The present study aimed to investigate, on molecular basics, the nephroprotective activity of azilsartan on renal IR injury in rats. Rats were assigned into four groups: (1) Sham group, (2) Azilsartan group, (3) IR group, and (4) IR/Azilsartan-treated group. Histological examination and renal function were evaluated. Levels of KIM-1, HMGB1, caspase 3, GPX, SOD, NF-κB, and p53 proteins were investigated using ELISA. mRNA levels of *IL-1β*, *IL6*, *IL10*, *TNF-α*, *NF-κB*, *p53*, and *bax* were assessed by qRT-PCR. Expression of p38, JNK, and ERK1/2 proteins was investigated by Western blotting. IR injury resulted in tissue damage, elevation of creatinine, BUN, KIM-1, HMGB1, caspase 3, NF-κB, and p53 levels, decreasing GPX and SOD activities, and up-regulation of *NF-κB*, *IL-1β*, *IL6*, *TNF-α*, *p53*, *and bax* genes. Furthermore, it up-regulated the expression of phosphorylated/total ratio of p38, ERK1/2, and JNK proteins. Interestingly, treatment of the injured rats with azilsartan significantly alleviated IR injury-induced histopathological and biochemical changes. It reduced the creatinine, BUN, KIM-1, HMGB1, caspase-3, NF-κB, and p53 levels, elevated GPX and SOD activities, down-regulated the expression of *NF-κB*, *IL-1β*, *IL6*, *TNF-α*, *p53*, *and bax* genes, and up-regulated *IL10* gene expression. Furthermore, it decreased the phosphorylated/total ratio of p38, ERK1/2, and JNK proteins. Azilsartan exhibited nephroprotective activity in IR-injured rats via its antioxidant effect, suppression of inflammation, attenuation of apoptosis, and inhibition of HMGB1/NF-κB/p38/ERK1/2/JNK signaling pathway.

## 1. Introduction

Renal ischemia/reperfusion (IR) injury is a condition characterized by a sudden blood flow impairment to the kidney. It is a prevalent event after kidney transplantation that can lead to delayed graft function, acute kidney injury (AKI), kidney rejection, and long-term risk of graft damage [1]. During renal IR injury, the ischemic damaged tissue produces an excessive amount of free radicals, which impair the mitochondrial electron transport chain, leading to the depletion of ATP and elevation of the intracellular calcium [2]. Additionally, the reperfusion phase can exacerbate the oxidative stress condition with the reactive oxygen species (ROS) generation, resulting in oxidative damage to DNA and proteins, which further initiates apoptotic signaling pathways and cell death [3]. Renal tubular cell apoptosis is also one of the most important pathophysiological processes during IR injury of the kidney [4]. Alteration of apoptotic, oxidative stress, and angiogenic signaling pathways plays an important part in cell survival, tissue restoration, and death [5,6,7,8]. Several antioxidant enzymes, including superoxide dismutase (SOD) and glutathione peroxidase (GPX), oppose the deleterious effect caused by ROS [9,10], thus targeting this pathway could be a beneficial strategy in protecting against tissue damage during IR injury.

Additionally, the initiation of oxidative stress is usually connected with robust inflammation that contributes to the severity of renal damage [11]. Renal IR injury is a systemic inflammatory disease caused by a cascade of pro-inflammatory mediators released from damaged kidney or blood cells. Cytokines, which are critical mediators of immune responses and inflammatory reactions, are implicated in numerous biological processes [12,13,14,15]. Tumor necrosis factor α (TNF-α) and Interleukin 6 (IL-6) are pro-inflammatory mediators involved in renal dysfunction during IR injury [16]. Moreover, activation of nuclear factor-κB (NF-κB) functions a crucial role in numerous pathological processes, including the pathogenesis of IR injury that causes acute renal failure [17,18].

The high mobility group (HMGB1) is a nucleoprotein that is linked to various biological processes, including DNA replication, autophagy, and apoptosis. Under stress conditions, it is translocated to the cytoplasm from the nucleus to be excreted into the extracellular extent and initiate immune response [19]. HMGB1 is a pro-inflammatory factor that acts as a damage-associated molecular pattern molecule activating the innate immune response by triggering numerous receptors on the plasma membrane [20,21]. Its pro-inflammatory role is implicated in a broad range of kidney disorders. It is highly expressed during IR injury, and its upregulation is correlated with injury severity [22]. Additionally, the renal oxidative stress has been accompanied by p38 mitogen-activated protein kinase (p38 MAPK), activation of c-jun N terminal kinase (JNK), reperfusion-induced necrosis, and extracellular signal-regulated kinases (ERK) activation, which can lead to several events, including mitochondrial death and cell apoptosis [23,24].

Moreover, the renin-angiotensin system (RAS) activation and elevation of angiotensin II levels participate in the progression of IR injury. Angiotensin II causes renal vessel constriction, oxidative stress initiation, and apoptosis induction [3,25]. Angiotensin II receptor blockers were described to exhibit protective effects against IR injury of the kidney [26,27].

Screening for new curative potentials of existing candidates has received a lot of attention [28,29,30,31,32,33]. Azilsartan is Food and Drug Administration (FDA)-approved as an antihypertensive candidate for adults. It is a member of angiotensin receptor blockers (ARBs), but it contains an oxo-oxadiazole ring which is not found in any of the clinically approved ARBs [34]. ARBs family has been investigated for its protective effects during IR injury [35,36]. Previously, azilsartan exerted protective effects against cerebral and myocardial IR injuries [37,38]. Additionally, azilsartan attenuated LPS-induced inflammation and oxidative stress in U937 macrophages and lung injury through the modulation of Nr2/HO-1 pathway [39,40]. Furthermore, it showed promising activity in acute myocardial infarction [41] and exerted a potential protective effect against cisplatin-induced cytotoxicity [42]. The present study aimed to examine the effect of azilsartan on renal IR injury in rats and the possible molecular mechanisms implied this effect by inspecting apoptosis and HMGB1/NF-κB/p38/ERK1/2/JNK signaling pathways.

## 2. Materials and Methods

### 2.1. Experimental Model

Animal care and study protocols were preceded according to The Declaration of Helsinki’s guidelines and endorsed by the Research Ethics Committee of Minia University, Egypt (ES08-2021). Animals were obtained from the National Research Center (Giza, Egypt), housed in separate cages, fed standard commercial pellets, supplied with fresh drinking water, and kept in a natural environment (12 h light/dark cycles). Renal IR injury was conducted as described before [17]. Pentobarbital sodium (50 mg/kg) was used intraperitoneally to anesthetize animals. To fully explore bilateral renal veins and arteries, a midline laparotomy was implemented. A non-invasive microvascular clamp was used to occlude the left renal pedicle for 30 min. Then, the clamp was loosened. After 48 h, rats were sacrificed to collect renal tissue and serum. Kidney was divided into three portions: The first portion was put in neutral buffered formaldehyde (10%) to be used for histological studies, the second and third tissue portions were kept at −80 °C directly for further protein and RNA analysis.

### 2.2. Experimental Design

Male Wistar rats were randomly distributed into 4 groups, each of ten rats. The first group (sham group), in which rats orally received carboxy methyl cellulose (CMC) daily for 9 days. The second group (azilsartan group) orally received 4 mg/kg of azilsartan daily [38] for 9 days. The third group (IR group) was the untreated IR group, where rats received CMC daily for 9 days. Then, they underwent 30 min renal ischemia on day 7, followed by 48 h reperfusion. The fourth group (IR/azilsartan group) is the treated IR group, where rats were pretreated orally with 4 mg/kg azilsartan dissolved in carboxy methyl cellulose (CMC) starting 7 days prior to the renal ischemia till the end of reperfusion (the end of the experiment) [38]. The experiment lasted for 9 days whereas, the renal ischemic procedures were performed on day 7.

### 2.3. Kidney Function Evaluation

Assay kits for creatinine and blood urea nitrogen (BUN) (Randox, London, UK) were utilized according to the manufacturer’s instructions to spectrophotometrically estimate kidney functions.

### 2.4. ELISA Techniques

Kidney tissues (500 mg) were homogenized in 500 μL of PBS on ice after cutting into small pieces. The supernatant was collected and analyzed after centrifugation at 5000 rpm for 15 min. Activities of GPX and SOD were determined according to the manufacturer’s instructions of the kits (#MBS744364 and #MBS036924, respectively, MyBioSource, San Diego, CA, USA), respectively. HMGB-1 tissue homogenate content was assessed using ELISA determination kit (#E-EL-R0505, Elabscience, Houston, TX, USA). Tissue homogenate caspase-3 and p53 proteins were investigated according to the manufacturer’s instructions for the kits (#CSB-E08857r and #CSB-E08336r, respectively, Cusabio, Houston, TX, USA). Tissue homogenate NF-κB protein was measured according to the manufacturer’s instructions for the kits (#MBS2505513, MyBioSource, San Diego, CA, USA).

Serum Kidney injury molecule-1 (KIM-1) was determined according to the manufacturer’s instructions using the corresponding kits (#CSB-E08808r, Cusabio, Houston, TX, USA).

### 2.5. Quantitative Real-Time Polymerase Chain Reaction

From renal tissue samples, total RNA was isolated according to the Qiagen RNA extraction kit (Hilden, Germany) instructions. By real-time qPCR, the expression of *IL-1β*, *IL6*, *TNF-α*, *NF-κB*, *p53*, and *bax* genes was investigated. As an internal control, *Glyceraldehyde 3-phosphate dehydrogenase (GAPDH)* was utilized [43]. mRNA was quantified using the Rotor-Gene 6000 Series Software 1.7. Primer sequences were obtained from National Centre for Biotechnology Information (NCBI), as shown in Table 1. Qiagen one-step RT-PCR (Qiagen, Hilden, Germany) was utilized for the experiment. Each reaction contained 1× buffer, total RNA (100 ng), 400 μM each of dNTP, 0.6 μM forward and reverse primers, and enzyme mix (2 μL). The experiment was performed under the following conditions: 35 cycles of denaturation step (25 s) at 95 °C, primers annealing (30 s) at 58 °C, and polymerization steps (20 s) at 72 °C.

A melting curve analysis was performed between 60–95 °C at 1 °C intervals, using the SYBR Green fluorescent dye, with the Rotor-Gene 6000 Series Software 1.7 to distinguish the obtained amplified mixture with the avoidance of contamination and to eliminate the production of non-specific compounds. Each sample was performed in triplicate during the experiment, and the average cycle threshold (Ct) was calculated. The target gene expression was calculated for each group relative to the untreated sham group after normalization to *GAPDH* expression.

### 2.6. Western Blotting Analysis

The expression of phosphorylated and total forms of p38, ERK 1/2, and JNK proteins was detected utilizing sodium dodecyl sulfate-polyacrylamide gel electrophoresis (SDS-PAGE) analysis.

To extract proteins from renal tissue samples, RIPA lysis buffer was utilized. The composition of RIPA lysis buffer was as follows: Sodium deoxycholate (0.5%), NaCl (150 mM), SDS (0.1%), PMSF (1 mM), Tris-Cl (50 mM), pH 7.5, and Nonidet P-40 (1%), enriched with the complete protease inhibitor cocktail (Roche, Mannheim, Germany). To estimate the protein concentration, the Bradford method was used [44]. At first, SDS-PAGE (15% acrylamide) was used to separate protein samples (30 μg). Then, proteins were transmitted to a Hybond™ nylon membrane (GE Healthcare) followed by incubation in Blocking Solution at room temperature for 1 h. At 4 °C, membranes were incubated overnight with antibodies of p-p38 (#9211, Cell Signaling Technology, Danvers, MA, USA), p38 (#9212, Cell Signaling Technology, Danvers, MA, USA), p-ERK1/2 (#ab201015, abcam, Cambridge, UK), ERK1/2 (#F54025, NSJ Bioreagents, San Diego, CA, USA), p-JNK (#NB100-82009, Novusbio, Littleton, CO, USA), and JNK (#sc-7345, Santa Cruz Biotechnology, Inc., Dallas, TX, USA) diluted (1:1000) with PBS. A 30–60 min washing period was utilized for membranes followed by an incubation with the diluted (1:1000 in PBS) HRP-conjugated secondary antibody (New England Biolabs, Hertfordshire, UK) for 1 h at room temperature [45]. An enhanced chemiluminescence kit (GE Healthcare, Little Chalfont, UK) was utilized to detect immunoreactive proteins by a luminescent image analyzer (LAS-4000, Fujifilm Co., Tokyo, Japan), according to the manufacturer’s instructions. β-actin, used as an internal control, was detected by β-actin antibody (New England Biolabs) (1:1000). Electroblotting and electrophoresis were carried out in a Bio-Rad Trans-Blot SD Cell apparatus (Bio-Rad, Hercules, CA, USA) using a discontinuous buffer system. Using The Image Processing and Analysis Java (ImageJ, ImageJ bundled with Java 8, 1.8.0_172, Bethesda, Maryland, USA, 1997) program, the densitometric analysis was achieved. Data were obtained relative to the untreated sham group after normalization to the corresponding β-actin levels.

### 2.7. Histological Examination

In 10% formaldehyde, kidney tissue sections were fixed, then, dehydrated in ascending levels of ethanol and inserted in paraffin. Hematoxylin-eosin (H&E) was used to stain the sections. Then, the histopathological changes were observed using the optical microscope (Leica DMRBE) with the DP Controller software. At least three investigators examined the samples who were blind to pathological information. Tissue sections were evaluated for structural changes in tubules (injury and necrosis), interstitial structural changes, and glomerular changes. The scoring system was performed semi-quantitatively in terms of tubulointerstitial damage as follows: 4 = 76–100%, 3 = 46–75%, 2 = 26–45%, 1 = 0–25%, and 0 = not at all [46].

### 2.8. Statistical Analysis

Mean ± standard deviation (SD) was used to express the data. To analyze the differences of multiple comparisons, one or two-way analysis of variance (ANOVA) followed by post hoc Dunnett test were performed utilizing Excel software (Microsoft, Redwood, WA, USA) and GraphPad Prism 9 statistical software (GraphPad, La Jolla, CA, USA). When the probability *p* values < 0.05, differences were considered significant.

## 3. Results

### 3.1. Creatinine and BUN Serum Levels

**Figure 1:**
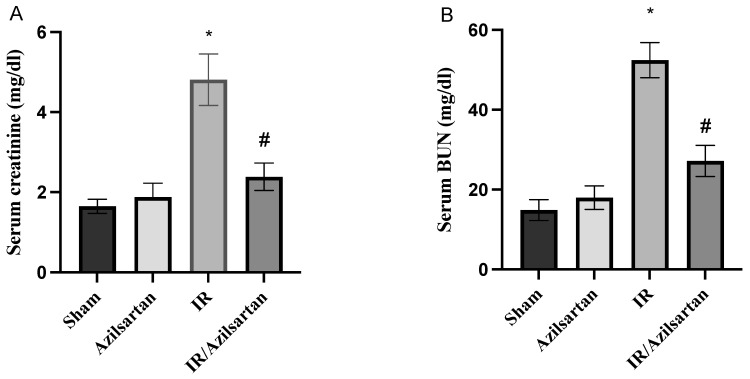
Creatinine (**A**) and BUN (**B**) serum levels. Bars represent mean ± SD. Followed by post hoc Dunnett test, significant difference was analyzed by one-way ANOVA test, where * *p* < 0.001, compared to the sham group, and # *p* < 0.001, compared to the IR group. BUN; blood urea nitrogen, SD; standard deviation, IR; ischemia reperfusion.

Creatinine and BUN serum levels were significantly increased to 4.81 ± 0.64 mg/dL and 52.4 ± 4.39 mg/dL in the IR group, respectively, when compared to the sham group. While the IR/azilsartan group showed a significant (*p* < 0.001) decrease in serum levels of both parameters to 2.39 ± 0.34 mg/dL and 27.19 ± 3.89 mg/dL, when compared to the IR group, as shown in Figure 1A non-significant difference (*p* > 0.05) was found in serum creatinine and BUN levels between the sham group and the azilsartan-treated group.

### 3.2. Levels of KIM-1, HMGB-1, Caspase 3, GPX, SOD, NF-κB and p53

**Figure 2:**
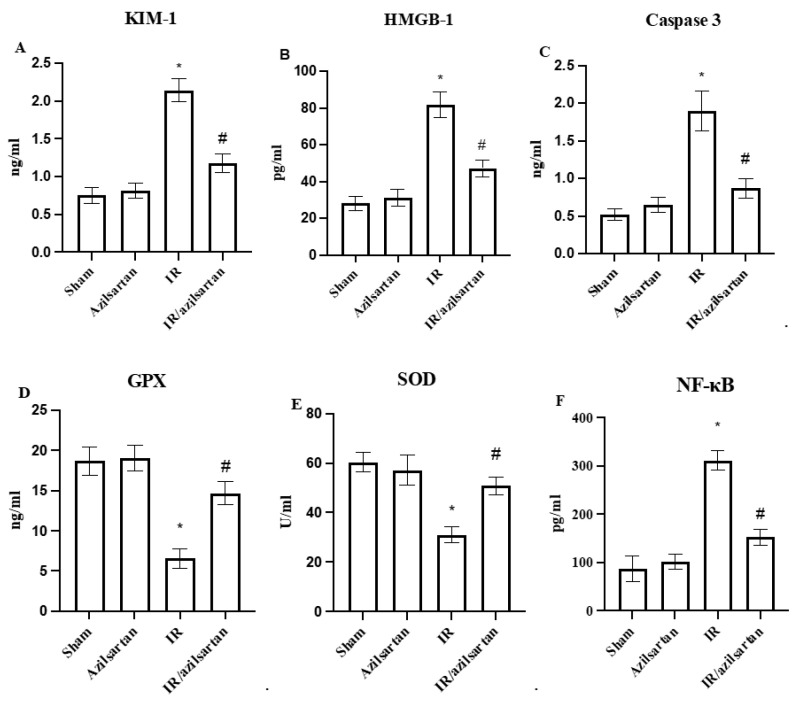

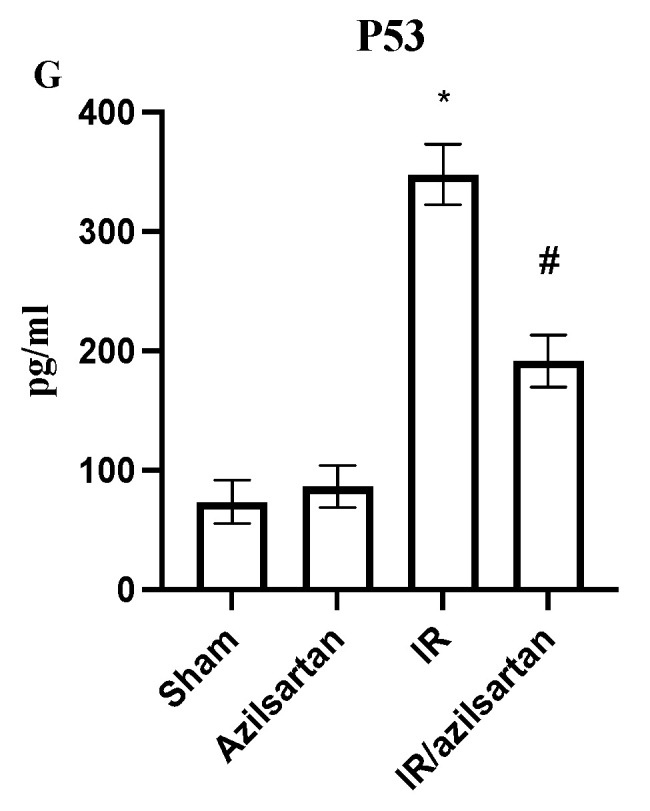
Levels of KIM-1 (**A**), HMGB-1 (**B**), caspase 3 (**C**), GPX (**D**), SOD (**E**), NF-κB (**F**) and p53 (**G**). Bars represent mean ± SD. Followed by post hoc Dunnett test, significant difference was analyzed by one-way ANOVA test, where * *p* < 0.001, compared to the sham group, and # *p* < 0.001, compared to the IR group. KIM-1; kidney injury molecule-1, GPX; glutathione peroxidase, SOD; superoxide dismutase, NF-κB; nuclear factor-κB, SD; standard deviation, IR; ischemia reperfusion.

As shown in Figure 2, when compared to the sham group, the IR group rats showed a significant (*p* < 0.001) increase in KIM-1, HMGB-1, caspase 3, NF-κB, and p53 protein levels, while levels of GPX and SOD were substantially (*p* < 0.001) decreased. Treatment of the injured rats with azilsartan decreased (*p* < 0.001) levels of KIM-1, HMGB-1, and caspase 3 to 1.17 ± 0.12 ng/mL, 47.20 ± 4.6 pg/mL, and 0.87 ± 0.12 ng/mL, respectively, when compared to the IR group. Additionally, levels of GPX and SOD were significantly (*p* < 0.001) increased in the IR/azilsartan group to 14.67 ± 1.44 ng/mL and 50.91 ± 3.59 U/mL when compared to the IR group. Furthermore, the NF-κB and p53 protein levels were significantly (*p* < 0.001) diminished in the IR/azilsartan group to 152.4 ± 16.9 pg/mL and 191.8 ± 21.8 pg/mL, respectively, when compared to the IR group. There was no significant difference (*p* > 0.05) in levels of all measured parameters observed in the azilsartan-treated group, when compared to the sham group.

### 3.3. Expression of IL-1β, IL6, IL10, TNF-α, NF-κB, p53, and Bax Genes

**Figure 3:**
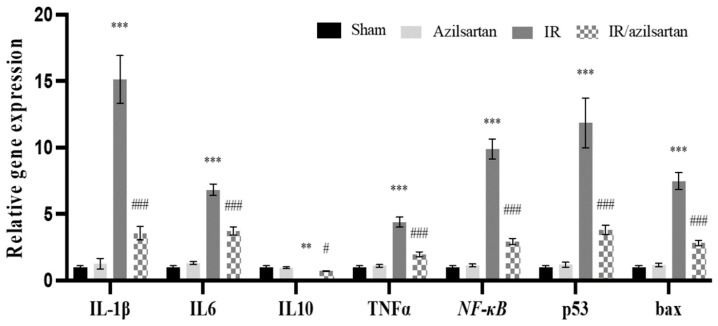
The renal expression of *IL-1β*, *IL6*, *IL10*, *TNF-α*, *NF-κB*, *p53*, and *bax* genes. Quantitative RT-PCR was used to investigate the gene expression of different groups. Expression was represented relative to the untreated sham group and normalized to the corresponding *GAPDH* gene expression. Bars represent mean ± SD. Followed by post hoc Dunnett test, significant difference was analyzed by two-way ANOVA test, where **; *p* < 0.01, ***; *p* < 0.001, compared to the sham group, and #, *p* < 0.05; ###, *p* < 0.001, compared to the IR group. IL; interleukin, TNF-α; tumor necrosis factor-α, NF-κB; nuclear factor-κB, SD; standard deviation, IR; ischemia reperfusion.

To investigate the effect of azilsartan on IR injured rats, mRNA levels of *IL-1β*, *IL6*, *TNF-α*, *NF-κB*, *p53*, and *bax* were examined in renal tissues of the different groups. As shown in Figure 3, a significant (*p* < 0.001) elevation in *IL-1β*, *IL6*, *TNF-α*, *NF-κB*, *p53*, and *bax* genes expression was observed in the IR group rats, while the expression of *IL10* gene was significantly (*p* < 0.01) decreased, when compared to the sham group.

When compared to the IR group, the IR/azilsartan group showed significant (*p* < 0.001) decrease in *IL-1β*, *IL6*, *TNF-α*, *NF-κB*, *p53*, and *bax* genes expression after normalization to the internal control *GAPDH*. mRNA levels of *IL10* were significantly (*p* < 0.05) increased in the IR/azilsartan group, when compared to the IR group. Moreover, mRNA levels of all investigated genes showed no significant difference (*p* > 0.05) between the sham group and the azilsartan group.

### 3.4. Expression of p38 MAPK, ERK1/2, and JNK Proteins

To further investigate the effect of azilsartan during IR injury and to explain the above-mentioned obtained effects, the expression of phosphorylated and total p38 MAPK, ERK 1/2, and JNK proteins was examined using Western blotting.

**Figure 4:**
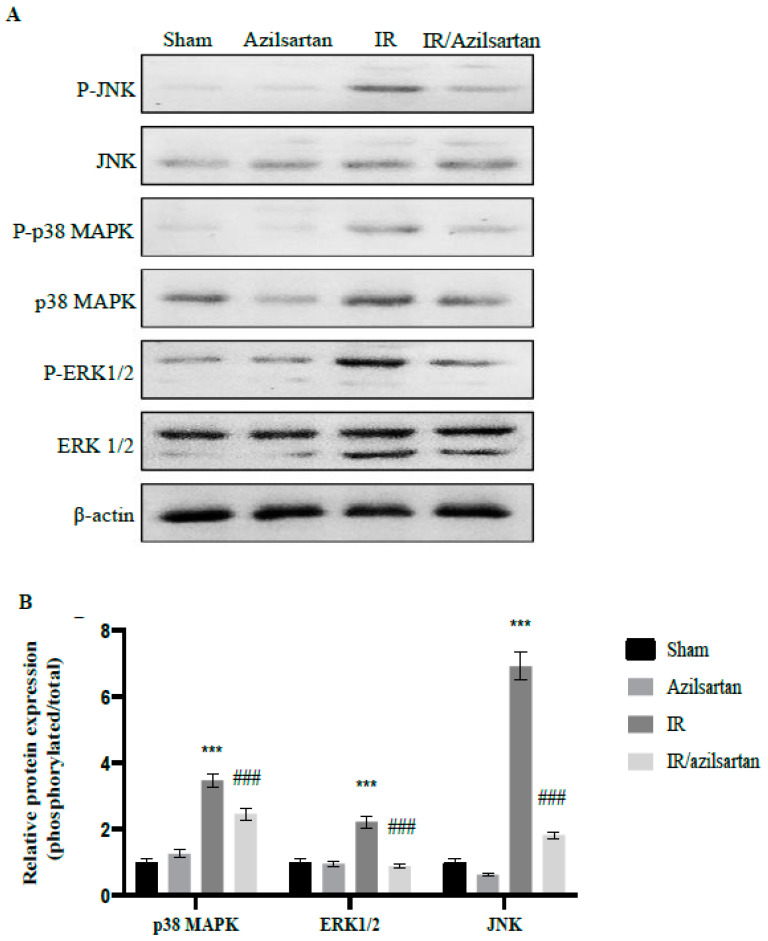
Effect of azilsartan on the expression of p38 MAPK, ERK1/2, and JNK proteins. (**A**) Representative Western blots of phosphorylated and total p38 MAPK, ERK1/2, JNK, and β-actin proteins for different groups. (**B**) Expressions of phosphorylated/total ratio of proteins were densitometrically expressed as fold change, using bands in (**A**), relative to that of sham control rats, after normalization to the corresponding β-actin. Bars represent mean ± SD. Followed by post hoc Dunnett test, significant difference was analyzed by two-way ANOVA test, where ***; *p* < 0.001, compared to the sham group, and ### *p* < 0.001, compared to the IR group. p38 MAPK; Mitogen Activated Protein Kinase, ERK1/2; extracellular signal-regulated protein kinase, c-JNK; Jun N terminal kinase, SD; standard deviation, IR; ischemia reperfusion.

In Figure 4, a significant (*p* < 0.001) renal elevation of phosphor/total ration of p38 MAPK, ERK1/2, and JNK proteins was observed in IR untreated rats compared to sham rats after normalization to *β*-actin. Remarkably, treating IR-injured rats with azilsartan led to significant (*p* < 0.001) down-regulation of phosphor/total ratio of p38 MAPK, ERK1/2, and JNK proteins, when compared to the IR group rats.

### 3.5. Histopathological Examination

**Figure 5:**
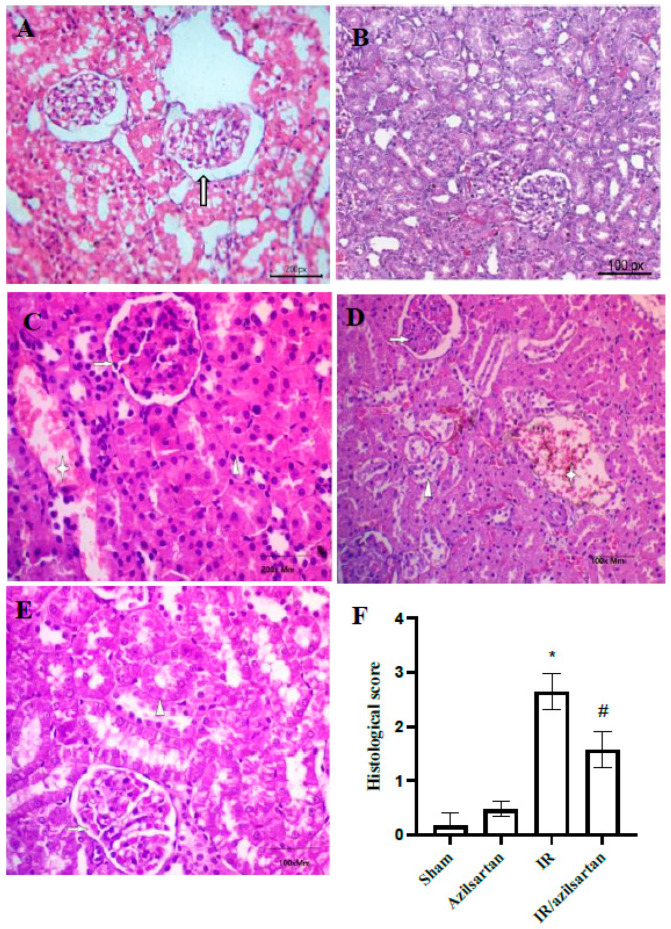
Representative photomicrographs of rat kidney tissues of different groups. Hematoxylin-eosin was used to stain kidney tissues. (**A**) The sham group (magnification; ×200), (**B**) the azilsartan-treated group (magnification; ×100), (**C**,**D**) the IR group (magnification; ×200 & ×100, respectively), and (**E**) the IR/azilsartan-treated group (magnification; ×100). (**F**) Histological score of kidney damage. Bars represent mean ± SD. Followed by post hoc Dunnett test, significant difference was analyzed by one-way ANOVA test, where * *p* < 0.001, compared to the sham group, and # *p* < 0.01, compared to the IR group. SD; standard deviation, IR; ischemia reperfusion.

For renal histological changes examination, tissues were evaluated using H&E staining to confirm the effect of azilsartan. As shown in Figure 5, (A) the sham group showed normal glomerular tuft with surrounded tubules and normal glomerular basement membrane (arrow), (B) the azilsartan-treated group showed normal tufts, normal interstitium, and no histopathological alterations, compared to rats of the sham group, as shown in Figure 5G. (C) The IR group showed congested tufts (arrow), mild tubular injury with hemorrhage (asterix) and normal interstitium (arrowhead), (D) the IR group showed retracted tufts (arrow), mild tubular necrosis (arrowhead) and interstitial hemorrhage (asterix), and (E) the IR/azilsartan group showed normal tufts (arrow), focal tubular injury (arrowhead) and normal interstitium (asterix).

## 4. Discussion

Renal IR injury is the primary reason for AKI, which contributes to elevated morbidity and mortality rate. Although the pathophysiology of renal IR injury is not well understood, in the progression of kidney failure, multiple mechanisms are involved [3]. Angiotensin II is a powerful intrarenal vasoconstrictor that induces oxidative stress [47]. It was reported that the oxidative stress indices increase, and renal IR injury were relatively correlated with the angiotensin II levels [48,49]. Therefore, targeting angiotensin II could be beneficial in attenuating renal IR injury. Therefore, various studies have examined the potential activity of ARBs against IR injury, for example, fimasartan and losartan attenuated inflammatory and apoptotic pathways in IR injury via modulating TNFα, IL-1β, and IL6 [50,51]. In addition, valsartan has shown protective activity in IR injury via NF-kB pathway inhibition [35]. Moreover, telmisartan modulated nitric oxide and TNFα during renal IR injury [52].

Looking for new pharmacological activities for novel agents [53,54,55,56] and repurposing drugs [57,58,59] have become a remarkable approach [14,60,61,62]. Azilsartan is a member of ARBs and has been used in the market as an antihypertensive drug [34]. It has been shown to reduce kidney damage and improve glycemic status in diabetic rats [63]. Moreover, in hypertensive obese rats, it decreased cardiovascular and renal injury [63]. Moreover, it exhibited neuroprotective and cardioprotective activities against cerebral and myocardial ischemia [37,38]. Furthermore, it targeted the vascular endothelial growth factor (VEGF) pathway during renal IR injury [27]. Additionally, via the NF-kB/IL-6/JAK2/STAT3 pathway inhibition in breast cancer cells, we recently demonstrated the anticancer activity of azilsartan [64]. Herein, we investigated the effect of azilsartan on apoptosis, ERK/JNK and NF-κB pathways during renal IR injury in rats.

The type 1 transmembrane protein, KIM-1, is only expressed on the proximal tubules in response to various toxins or pathophysiological states. It was proved to be a specific, sensitive kidney injury marker, and an indicator of the outcome as well [65]. Our findings showed that treatment of the IR-injured rats with azilsartan exerted a notable improvement in their renal function, which was represented by decreasing the levels of creatinine, BUN, and especially KIM-1. The histopathological examinations confirmed our hypothesis that renal IR-injured rats treated with azilsartan showed normal glomerulus without inflammatory infiltrates.

The ROS generation during IR injury results in the deleterious effects of IR injury on the cell, with subsequent inflammatory response initiation and AKI development [66]. Additionally, the generation of ROS activates the cell death signaling pathways such as apoptosis and necrosis [67,68]. In the present study, GPX and SOD activities were found to be decreased during IR injury, but after azilsartan treatment, they were notably elevated, which suggests the antioxidant activity of azilsartan, which also has been previously observed through the repression of angiotensin and the oxidative stress [40,69].

It was reported that HMGB1 could link to and stimulate receptors for advanced glycation end products with further stimulation of the ERK, JNK, p38, and NF-κB pathways [70] which, upon activation, lead to the pro-inflammatory cytokines release, including TNF-α, IL-1β, and IL6, and the inflammatory response initiation [6,71,72]. ERK, JNK, and p38, which are also known as stress kinases, are stimulated in response to stress stimuli such as inflammatory cytokines, ROS-mediated oxidative stress, and renal IR injury, which further mediates fibrosis, inflammation, and apoptosis [73,74]. Several studies documented the activation of ERK, JNK, p38 proteins during renal ischemia, thus, targeting this pathway could be a protective approach during IR injury [75,76,77]. Our findings revealed the high expression of NF-κB and HMGB1 proteins and mRNA levels of *NF-κB*, *IL-6*, *IL-1β*, and *TNF-α* during IR injury, in addition to the increased phosphorylation of ERK1/2, JNK, and p38 proteins, which suggested the activation of these pathways during IR. While IR-injured rats treated with azilsartan showed a notable decrease in HMGB1 and NF-κB proteins, and mRNA levels of *NF-κB*, *IL-6*, *IL-1β*, and *TNF-α*, in addition to the suppression in the activation and phosphorylation of ERK1/2, JNK, and p38 proteins.

It has been proposed that the activated p38/ERK/JNK signaling pathway contributes directly to cell cycle arrest and p53-mediated apoptosis in cardiomyocytes after myocardial infarction and in Friend murine erythroleukemia virus-transformed cell line [78,79,80]. In the present study, the p53 (protein and mRNA) expression, mRNA levels of *bax*, and the activity of caspase-3 were elevated, which suggest the induction of apoptosis and cell death during IR injury. At the same time, azilsartan treatment down-regulated the expression of p53 (protein and mRNA) and mRNA *bax* levels and suppressed the activity of caspase-3, suggesting the anti-apoptotic and protective potential of azilsartan during IR injury.

IL-10, which limits the immune response to external stimuli or pathogen, is a potent anti-inflammatory cytokine, thus, maintaining normal tissue hemostasis and preventing the damaging effect [81]. In the present study, the *IL-10* gene expression was found to be suppressed during IR injury, while after azilsartan treatment, its expression was elevated.

The present study took a closer look at the molecular targets of azilsartan during renal IR injury. However, for more recognition of the molecular mechanisms by which azilsartan might extend its valuable renal protection during renal IR injury, future work is required, and additional inspections using different doses of azilsartan are necessary to increase the awareness with regard to the competency of our findings to be clinically applicable.

## 5. Conclusions

This study demonstrated that azilsartan, which is an FDA-approved antihypertensive drug, exerted promising nephroprotective activity during IR injury in rats via its antioxidant, anti-inflammatory, and anti-apoptotic effects. Our findings revealed that azilsartan showed antioxidant activity by increasing the activity of GPX and SOD. Furthermore, it attenuated inflammation by increasing the expression of *IL-10* gene, targeting HMGB1/NF-κB/p38/ERK/JNK signaling pathways and reducing the mRNA levels of *NF-κB*, *IL-6*, *IL-1β*, and *TNF-α*. Additionally, it suppressed apoptosis by attenuating the levels of p53 and caspase-3 proteins and decreasing mRNA levels of *p53* and *bax* genes.

## Figures and Tables

**Table 1:** Sequences of the primers.

Primer	Sequence of the Primer
*IL-1β*	Forward: 5′-CCTATGTCTTGCCCGTGGAG-3’ Reverse: 5′-CACACACTAGCAGGTCGTCA-3′.
*IL6*	Forward: 5′-CCTACCCCAACTTCCAATGCT-3′ Reverse: 5′-GGTCTTGGTCCTTAGCCACT-3′.
*IL10*	Forward: 5′-CTGGCTCAGCACTGCTATGT-3′ Reverse: 5′-GCAGTTATTGTCACCCCGGA-3′.
*TNFα*	Forward: 5′-GGAGGGAGAACAGCAACTCC-3′ Reverse: 5′-GCCAGTGTATGAGAGGGACG-3′.
*NF-κB*	Forward: 5′-TCAACATGGCAGACGACGATCC-3′ Reverse: 5′-GAAGGTATGGGCCATCTGTTGAC-3′.
*P53*	Forward: 5′-AGCGACTACAGTTAGGGGGT-3′ Reverse: 5′-ACAGTTATCCAGTCTTCAGGGG-3′.
*Bax*	Forward: 5′-CGTCTGCGGGGAGTCAC-3′ Reverse: 5′-AGCCATCCTCTCTGCTCGAT-3′.
*GAPDH*	Forward: 5′-AACCTGCCAAGTATGATGACATCA-3′ Reverse: 5′-TTCCACTGATATCCCAGCTGCT-3′.

## Data Availability

All data are fully available and included in the manuscript.
